# Dosimetric assessment of a novel metal artifact reduction method in CT images

**DOI:** 10.1120/jacmp.v14i1.4027

**Published:** 2013-01-07

**Authors:** Maria F. Spadea, Joost Verburg, Guido Baroni, Joao Seco

**Affiliations:** ^1^ Department of Experimental and Clinical Medicine University of Magna Graecia Catanzaro Italy; ^2^ Department of Radiation Oncology Havard Medical School and Massachusetts General Hospital MA USA; ^3^ Bioengineering Department Politecnico di Milano University Milano Italy; ^4^ Bioengineering Unit Fondazione CNAO Pavia Italy

**Keywords:** CT, metal artifact reduction, dose inaccuracies, IMRT

## Abstract

The aim of this study was to assess the ability of metal artifact reduction (MAR) algorithm in restoring the CT image quality while correcting the tissue density information for the accurate estimation of the absorbed dose. A phantom filled with titanium (low‐Z metal) and Cerrobend (high‐Z metal) inserts was used for this purpose. The MAR algorithm was applied to phantom's CT dataset. Static intensity‐modulated radiation therapy (IMRT) plans, including five beam angles, were designed and optimized on the uncorrected images to deliver 10 Gy on the simulated target. Monte Carlo dose calculation was computed on uncorrected, corrected, and ground truth image datasets. It was firstly verified that MAR methodology was able to correct HU errors due to the metal presence. In the worst situation (high‐Z phantom), the image difference, uncorrected ground truth and corrected ground truth, went from −4.4±118.8 HU to 0.4±10.8 HU, respectively. Secondly, it was observed that the impact of dose errors estimation depends on the atomic number of the metal: low‐Z inserts do not produce significant dose inaccuracies, while high‐Z implants substantially influence the computation of the absorbed dose. In this latter case, dose errors in the PTV region were up to 23.56% (9.72% mean value) when comparing the uncorrected vs. the ground truth dataset. After MAR correction, errors dropped to 0.11% (0.10% mean value). In conclusion, it was assessed that the new MAR algorithm is able to restore image quality without distorting mass density information, thus producing a more accurate dose estimation.

PACS numbers: 87.53.Bn, 87.55.K, 87.57.cp

## I. INTRODUCTION

Many metal artifact reduction (MAR) methods are becoming available and allow for improved CT image quality for radiotherapy planning and dose calculation. The most common approach is to disregard projections through metal objects and to interpolate for recovering missing data. Several authors investigated the possibility of using linear, cubic spline or wavelet interpolation on the individual projections, sometimes combined with multidimensional adaptive filtering for additional noise reduction and segmentation.^(^ ^1^ ^,^ ^2^ ^,^ ^3^ ^,^ ^4^ ^,^ ^5^ ^)^ Other methods aim to reduce the beam hardening effect and quantum noise in the image space.^(^ ^6^ ^,^ ^7^ ^)^ In some cases, different CT scan protocols have been proposed and tested to reduce image noise.^(^ ^8^ ^)^ Although evident improvements for volume of interest visualization and contouring have been reported, these algorithms do not account for the physical characteristics of the metal material that also influence dose computation in streaked regions of the CT image.

In this study, the dosimetric impact in intensity‐modulated radiation therapy (IMRT) of a novel metal artifacts reduction (MAR) algorithm, which was developed at Massachusetts General Hospital (Boston, USA),^(^ ^9^ ^)^ was tested. The novelty of this MAR method is that it accounts for either or both of the following: i) beam hardening effects (mostly relevant in low‐Z), and ii) photon starvations effects (mostly relevant in high‐Z). The image reconstruction was performed using a combination of the following methods: (1) to reduce beam hardening, a “physics correction” method was developed by correcting the intensity of only those projections passing through the implant; and (2) to account for missing angle projections due to photon starvation, an “iterative regularization” method was used with consistency conditions (i.e., mass conservation) and image smoothness constraints.

The analysis carried out in this phantom study was focused on performing image and dose analysis of the uncorrected and corrected datasets relative to a ground truth. The dosimetric impact of the developed MAR method on IMRT dose distributions, where the variable intensities across a beam profile are most susceptible to metal artifacts, was assessed.

## II. MATERIALS AND METHODS

**Figure 1:**
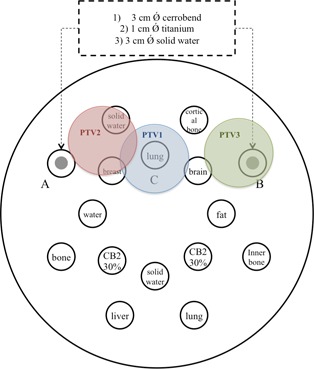
Cross‐sectional layout of the Gammex phantom filled with different materials. The phantom was scanned three times with three different inserts used: titanium, Cerrobend or solid water, in slot A and B.

The Gammex 467 phantom (Gammex Inc., Middleton, WI) was used in this work. It can include up to 16 different tissue‐equivalent materials. Figure 1 shows the cross‐sectional layout of the phantom that was scanned in three different configurations, as slot A and B included:

1. 1 cm diameter of titanium inserts ($\rho =4.540\;g/{\text{cm}}^{3}$);
2. 3 cm diameter of Cerrobend (Bolton Metals Products, Bellefonte, PA) inserts ($\rho =9.760\;\;g/{\text{cm}}^{3}$); and
3. 3 cm diameter of solid water insert ($\rho =1.004\;\;g/{\text{cm}}^{3}$).

**Figure 2:**
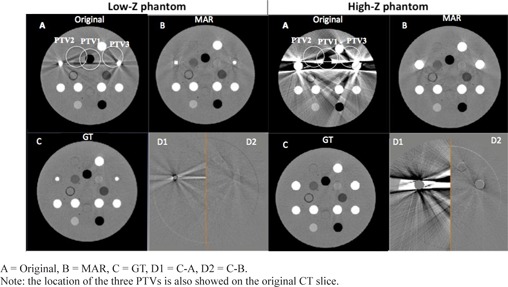
A = Original, B = MAR, C = GT, D1 = C−A, D2 =C−B. Note: the location of the three PTVs is also showed on the original CT slic Image differences for low‐Z and high‐Z phantom.

Configurations 1) and 2) represented the low‐Z and high‐Z phantom, respectively. In configuration 3) the solid water was manually segmented out and replaced by software by: i) 1 cm diameter of titanium, and ii) 3 cm diameter of Cerrobend densities, to define the ground truth (GT) of the images without artifacts. Three locations (see Fig. 1) of a cylindrical planning target volume, PTV, (4 cm diameter) were drawn to explore different areas of the CT volume. As it is shown in Figs. 1 and 2, PTV3 included portions of the metallic inserts, whereas PTV1 and PTV2 included areas with substantial artifacts in the uncorrected image but did not contain any portions of the metallic inserts.

A clinical scanner (GE LightSpeed RT16, GE Healthcare, Waukesha, WI) was employed to acquire and reconstruct volumetric image of the phantom with a CT range of −1024 to 3071. Acquisition parameters were: axial mode scanning, 2.5 mm slice thickness, 140 kVp, and automatic exposure control (AEC). Images were reconstructed with GE standard Filtered Back Projection.

The MAR algorithm^(^ ^9^ ^)^ was run to recover streak artifacts caused by metals with low‐Z and high‐Z. Static IMRT plans were designed for each location of PTV by setting five beam angles to cover the target with a nominal dose of 10 Gy. Beam angles were chosen to avoid the metal along their path in phantom (PTV1 and PTV2), as prescribed by AAPM Task Group 63.^(^ ^10^ ^)^ In the third plan, since the insert was included in the target, PTV3, the beam passed through the metal necessarily.

An in‐house Monte Carlo‐based procedure^(^ ^11^ ^)^ was used to calculate the dose distribution on all image volumes — uncorrected, corrected, and GT. The CT Hounsfield units (HUs) were converted to electron density according to the procedure described in Vanderstraeten et al.^(^ ^12^ ^)^

The analysis was focused on assessing the accuracy of MAR algorithm for image correction and dose calculations purposes. In the first case, voxel‐by‐voxel differences between corrected and GT and corrected and uncorrected image volumes were computed and compared in terms of HU. The mean difference and the standard deviation (std) were computed as a measure of central tendency and dispersion, respectively. In particular, the std amplitude was considered as the similarity index between uncorrected and corrected images relative to GT. To evaluate dose inaccuracies, dose volume histograms for each phantom were generated and analyzed in terms of dose received by the 95% ($D_{95}$), 98% ($D_{98}$) and 100% ($D_{100}$) of the PTV's volume. Both uncorrected and corrected datasets were compared to the GT to compute the relative accuracy error, as follows:

(1) $$Acc_{x}[\%]=\frac{\left|D_{x}-D_{xGT}\right|}{D_{xGT}}\cdot 100$$

with x=95, 98, 100.

Furthermore, the voxel‐by‐voxel difference (dose volume difference) was calculated for each PTV and both metals:

1. GT‐uncorrected, to quantify dose errors; and
2. GT‐corrected, to assess the correction performed by MAR algorithm.

These were presented in terms of color maps quantifying the errors. Wilcoxon‐Mann‐Whitney test was applied to assess the level of confidence of the findings.

## III. RESULTS

Results assessing MAR algorithm correction are shown in Fig. 2 for low‐ and high‐Z phantoms. Boxes D1 and D2 represent the difference between GT (C) vs. uncorrected (A) and GT vs. corrected (B), respectively.

By analyzing image difference histograms, it was found (mean ± std):

1. Low‐Z phantom, HU differences decreased from −0.8±34.51 HU (D1), to 0.2±8.5 HU (D2).
2. High‐Z phantom, HU differences decreased from −4.4±118.8 HU (D1) to 0.4±10.8 HU (D2).

Table 1 shows values of the accuracy at $D_{95}$, $D_{98}$, and $D_{100}$ relative to GT for the uncorrected and corrected dataset, respectively. Data are presented for the high‐Z phantom only, being the worst discrepancy for the low‐Z phantom in the uncorrected version below 0.3%. Results clearly show that the PTV dose computed on the corrected volume essentially matches the GT with a maximum error of 0.38%.

**Table 1:** Relative accuracy error between uncorrected vs. GT and corrected vs. GT at $D_{95}$, $D_{98}$, and $D_{100}$.

	*Uncorrected*		*Corrected*
		*PTV1*	
${\text{Acc}}_{95}$	4.26%		0.11%
${\text{Acc}}_{98}$	12.14%		0.11%
${\text{Acc}}_{100}$	23.56%		0.00%
		*PTV2*	
${\text{Acc}}_{95}$	2.01%		0.11%
${\text{Acc}}_{98}$	7.88%		0.11%
${\text{Acc}}_{100}$	25.70%		0.00%
		*PTV3*	
${\text{Acc}}_{95}$	3.32%		0.10%
${\text{Acc}}_{98}$	2.90%		0.38%
${\text{Acc}}_{100}$	5.71%		0.00%

**Figure 3:**
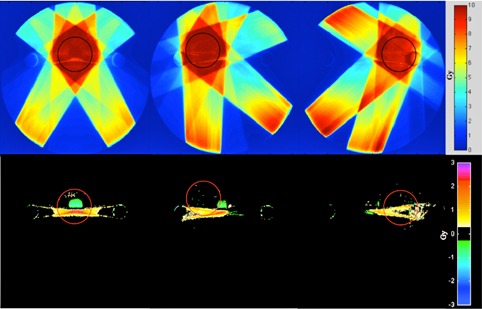
High‐Z phantom original dose distribution (upper panels) in PTV1 (left), PTV2 (middle), PTV3 (right); GT‐Original dose cube differences (lower panels) in PTV1 (left), PTV2 (middle), PTV3 (right). Color maps are scaled in Gy.

In Fig. 3, the cross section of the planned dose on the uncorrected phantom (upper panels) and of the dose difference (uncorrected GT) is shown. Appreciable discrepancies were found comparing voxel‐by‐voxel differences. Extreme values were: −2 to 4 Gy for PTV1 plan, −3 to 5 Gy for PTV2, and −3 to 4 Gy for PTV3, being the prescribed dose 10 Gy. These values were reduced to 0.5 Gy maximum in the corrected version.

Wilcoxon‐Mann‐Whitney test at 0.01% significance indicated no statistical differences ($p<10^{-8}$) between corrected and GT in terms dose computed on any PTV. The largest range difference was found in a small region around the metal in high‐Z phantom, PTV3, being about ± 0.5 Gy.

## IV. DISCUSSION & CONCLUSIONS

In this work, a novel method to correct CT images corrupted by metal artifacts was tested from a dosimetric point of view. The developed algorithms distinguished between i) beam hardening, and ii) photon starvation due to low‐Z and high‐Z metal implants. Titanium (low‐Z) and Cerrobend (high‐Z) were additional inserts used with the Gammex phantom for this purpose. High‐Z implants caused larger HU differences relative to low‐Z, when comparing the reconstructed images with the GT (std ≈ 120 HU versus std ≈ 35 HU). The MAR algorithms reduced the HU differences significantly to std ≈ 10 HU, with high‐Z and low‐Z having a residual error of the same order. By computing the maximum image difference, it was found 1050 HU and 250 HU for high‐Z and low‐Z, respectively, thus confirming that the local image corruption was much larger for Cerrobend.

Although in this paper the main aim was to measure the accuracy of the dose computed on the corrected images relative to the GT, it was also possible to observe and speculate the dose inaccuracies caused by the presence of metal artifacts. Dose errors were significant only for high‐Z phantom setting, causing discrepancies up to ≈ 23% in the dose received by the PTV volume. In this case, it was observed that the PTV1 had the highest overdosage errors because it was centered on the imaging artifact streaks (see Fig. 3). By comparing the dose voxel‐by‐voxel mismatches, up to 5 Gy were found. In particular, Fig. 3 shows that hot and cold spots occurred where streak artifacts are present rather than where the maximum nominal dose is optimized and delivered. In any circumstance studied, image correction performed through the MAR method led to accurate dose calculation, as reported by the comparison between corrected vs. GT. The maximum dose residual error after metal artifacts reduction (computed over whole voxels of the dose volume) was 5% of the prescribed dose. On average, the dose estimation on the PTV had a relative accuracy of 0.11%, much lower than the clinically accepted threshold for IMRT treatment (1%–2%).

These results confirm the proposed algorithm for metal artifacts recovery coming from low‐and high‐Z is accurate in a solid water phantom, and encourage the use of the procedure in the clinical framework of radiation therapy. Further studies are needed to investigate the role of MAR methods on dose calculation accuracy in clinical IMRT treatment planning.

## ACKNOWLEDGMENTS

This work was partly supported by a Fulbright fellowship awarded by the U.S.‐Italy Fulbright Commission.
